# Structural Retrofitting of Corroded Reinforced Concrete Beams Using Bamboo Fiber Laminate

**DOI:** 10.3390/ma14216711

**Published:** 2021-11-08

**Authors:** Paul Oluwaseun Awoyera, Tobechukwu Austin Nworgu, Balaji Shanmugam, Krishna Prakash Arunachalam, Iman Mansouri, Lenin Miguel Bendezu Romero, Jong-Wan Hu

**Affiliations:** 1 Department of Civil Engineering, Covenant University, Ota 112233, Nigeria; paul.awoyera@covenantuniversity.edu.ng (P.O.A.); nworgu.tobechukwu@lmu.edu.ng (T.A.N.); 2 Department of Civil Engineering, Kongu Engineering College, Perundurai 638060, India; er.shbalaji@gmail.com; 3 Department of Civil Engineering, Anna University, Chennai 600025, India; krishnaprakash3191@gmail.com; 4 Department of Civil Engineering, Birjand University of Technology, Birjand 9719866981, Iran; mansouri@birjandut.ac.ir; 5 Departamento Académico de Estructuras, Universidad Ricardo Palma, Lima 1801, Peru; lenin.bendezu@urp.edu.pe; 6 Department of Civil and Environmental Engineering, Incheon National University, Incheon 22012, Korea; 7 Incheon Disaster Prevention Research Center, Incheon National University, Incheon 22012, Korea

**Keywords:** concrete repair, sustainable materials, corrosion control, fiber laminate, structural failure, bamboo laminate

## Abstract

Corrosion creates a significant degradation mechanism in reinforced concrete (RC) structures, which would require a high cost of maintenance and repair in affected buildings. However, as the cost of repairing corrosion-damaged structures is high, it is therefore pertinent to develop alternative eco-friendly and sustainable methods. In this study, structural retrofitting of corroded reinforced concrete beams was performed using bamboo fiber laminate. Three reinforced normal weight concrete beams were produced, two of which were exposed to laboratory simulated corrosion medium, and the remaining one sample served as control. Upon completion of the corrosion cycle, one of the two corroded beams was retrofitted externally with a prefabricated bamboo fiber laminate by bonding the laminate to the beam surface with the aid of an epoxy resin. The three beams were subjected to loading on a four-point ultimate testing machine, and the loads with corresponding deflections were recorded through the entire load cycle of the beams. Finally, the mass loss of embedded steel reinforcements was determined to measure the effect of corrosion on the beams and the steel. The result showed that corroded beams strengthened with bamboo laminates increase the bearing capacity. Using a single bamboo laminate in the tensile region of the corroded beam increased the ultimate load capacity of the beam up to 21.1% than the corroded beam without retrofit. It was demonstrated in this study that the use of bamboo fiber polymer for strengthening destressed RC beams is a more sustainable approach than the conventional synthetic fibers.

## 1. Introduction

Reinforced concrete is a widely used composite construction material that employs steel usage due to its remarkable tensile properties that help in the overall performance of the composite material. Steel has strong tensile properties, which allows it to be used in reinforced concrete and makes its relationship with concrete a mutually beneficial one. However, steel corrosion compromises this relationship and threatens to negatively change this relationship [1]. Steel is a thermodynamically unstable human-made occurring metal that deteriorates to return itself to a more stable natural state via corrosion. Steel corrosion in RC structures causes significant losses to a nation’s economy. Losses of 4–5% of the gross national product (GNP) of industrial countries have been reported to have been lost dealing with corrosion costs [2,3]. 

Corrosion is launched on RC structures when damaging elements to steel such as CO_2_ and chlorine make their way to the steel reinforcement. Corrosion induces many concrete damages such as spalling, cracking, debonding; delamination; reduction of the yield, ductility, and steel reinforcement cross-sectional area; and premature failure [1,4]. Due to the high alkalinity of concrete, embedded steel is prevented from corrosion by forming a thin passive oxide film (Fe_2_O_3_) around the reinforcing steel. However, corrosive agents (CO_2_ and chlorine) eliminate the oxide film by penetrating through the voids formed in the concrete by either lowering the PH value of the concrete or attacking the oxide film. This allows the embedded steel to contact moisture and oxygen, consequently causing the steel to corrode [5,6].

Due to the need to keep structures safe and functional from structural deterioration, especially corrosion, various strengthening, and retrofitting techniques have arisen to solve such problems. The usage of fiber-reinforced polymer (FRP) composites to strengthen and reinforce structural elements has become increasingly popular over recent years over traditional strengthening methods (post-tensioning, section enlargement, tensile reinforcement of reinforced concrete elements using steel plate bonding, use of cement grout or ferro-cement, and employing shotcrete) [7]. FRPs are utilized as rebar reinforcement and external bond reinforcement for concrete, steel, masonry, and timber structures [8,9]. FRPs are composite materials consisting of high tensile strength fibers attached in a polymer resin matrix to a concrete surface with the help of epoxy resins and have several advantages that are responsible for their rapid growth in the construction industry, such as their light-weight, high strength-weight ratio, good fatigue resistance, high specific tensile strength, high corrosion resistance, and ease of application [8,10].

Synthetic or artificial fibers such as carbon, aramid, and glass, amongst many, are the most widely used fibers employed as FRP reinforcements in strengthening and retrofitting concrete structures [11]. The most popular amongst synthetic fibers are carbon fiber and is most commonly mentioned and used in previous studies and retrofitting applications. Its high tensile strength combined with a comparable elasticity modulus to steel gives this fiber its position amongst researchers and engineers [11,12]. Glass fiber is another popularly used fiber in retrofitting applications, although, its lower cost, high deformability, break resistance, and good impact properties compared to CFRP make it in good demand in strengthening and retrofitting applications. It has lower tensile strength and elasticity modulus when compared with CFRP [11,13]. Aramid fibers are also popular with advantages such as high modulus of elasticity, great dimensional stability, no melting point, good fatigue behavior, high electrical, and corrosion resistance [14,15]. Synthetic fibers have been reported to be hazardous to human health due to airborne fiber particles that are released during their production and handling. A common health issue is a dermatitis that is suffered by workers involved with glass fiber application and products. In addition, synthetic fibers are not easily reusable, recyclable, biodegradable after their service life, readily available, especially in rural regions and third world countries, and sustainable [11,16].

These problems have led to the exploration of viable alternatives of natural and renewable resources to replace these materials that are not environmentally friendly. Natural fibers are good substitutes to synthetic fiber composites, especially in strengthening and retrofitting materials in the construction industry [16]. The employment of natural fibers as composites has been in use for a couple of years. They include kenaf, grass reeds, wood fiber, wheat, oats, barley, hemp, flax, jute, rice husks, rye, sugar cane, bamboo Kane, straw, ramie, sisal, Raphia, etc. [17]. Another investigation [18] demonstrated that hybridization of natural fibers with steel fiber could offer higher mechanical performance of composite. Fabrication of laminate using kenaf, jute, and jute rope fibers for structural application has also been reported [16]. The shear strength of the developed laminate was comparable to that of carbon fiber-reinforced polymer. It has also been researched that long natural fibers exhibit better performance like the synthetic fibers.

Bamboo is an adequate option amongst natural fiber as a composite in strengthening and retrofitting concrete elements. Its high tensile strength; cheaper cost compared to CFRP, wood, and steel; rapid growth; and mechanical and durability properties are some of the characteristics that are making this plant fiber gain increasing interest with researchers and engineers [19,20,21,22,23].

Some past studies have investigated the use of natural fibers as a composite to strengthen reinforced concrete (RC) beams in flexure and shear [24,25,26,27]. However, studies covering the use of bamboo as a fiber or laminate in strengthening and retrofitting RC concrete beams have not been overly reported. For instance, Chin et al. [21] did a study on the potential of using bamboo fiber composite plate to strengthen RC beams in flexure. In the study, six RC beams were cast and tested, which included two control beams, two un-strengthened beams, and two strengthened beams, followed by measuring the tensile and flexural strength of BFCP. Using the four-point bending test, all the beams were tested to failure, and beams strengthened with BFCP were found to exhibit greater structural capacity strength compared to the un-strengthened beams. Chin et al. [22] investigated the structural behavior of RC beams that were with and without openings strengthened externally with bamboo fiber-reinforced composite (BFRC) plates, both in shear and flexure. The investigations showed that the openings affected the original beam capacity negatively, and strengthening of RC beams with epoxy-based BFRC plates increased its capacity back to 98% of the control beam. The study also showed that the BFRC plates diverted crack formation from the strengthened section and enhanced the beam’s ductility.

Thus, the use of bamboo can be seen as a natural solution to the structural performance of concrete. In the current study, bamboo fiber laminate is being used to strengthen the structural beam exposed to corrosion in a laboratory simulated corrosive medium. In this study, a composite material of epoxy resin reinforced with long unidirectional bamboo fibers, to be used to strengthen corroded RC beams in flexure, was developed. A tensile test was performed on the fabricated composite to determine its tensile strength following the ASTM standards. The strengthening extent of the bamboo fiber-reinforced polymer (BFRP) laminate on RC beam was determined experimentally by carrying out a four-point bending test on a BFRP-strengthened RC beam, with reference to an un-strengthened beam. Comparisons were drawn between the experimental results of both the un-strengthened and strengthened beams in terms of the load versus deflection plot. Assumptions adopted in this study were presented. The results and outcome of this research are expected to serve as a guide to concrete designers and constructors for solving problems relating to concrete member corrosion in practice.

### Significance of the Study

The corrosion phenomenon causes a greater volume of the rust in concrete composite; thus, it results in a most detrimental effect of cracking and spalling of the concrete cover due to tensile stresses developing [28,29]. Along with the aesthetics being affected, the mechanical performance and load capacity are also adversely affected by cracking. Literature reports show that, as the cracks become bigger, water permeation increases, allowing aggressive agents access to the reinforcements. The aggressive agents initiate the corrosion process with water and oxygen [30]. For steel corrosion to occur, certain conditions must be met: An electrolyte, a metallic connection, and a minimum of two steel locations of the steel rebar at different energy levels.

The use of the hybrid technique between near surface mount (NSM) and the external bond (EB) technique has shown to mitigate disadvantages of each technique as mentioned in the literature review, making it more desirable. Yet, concerning FRP composites, especially with natural fibers, limited research explores this technique. Though minimal studies exist relating to the use of strengthening techniques employed retrofitting beams with bamboo fiber, the studies which deal with it use the EB technique due to ease of use and lower costs.

Bamboo as a reinforcing material is utilized in various forms such as mats, fibers, powder, and strips, with strips shown to have the better cohesive ability on structural members. Yet, most studies explore bamboo as a reinforcing material in retrofitting structural members, primarily as fibers [21,22] and few to none on strips. However, numerous studies covered bamboo fibers [31,32,33].

## 2. Materials and Methods

The experimental study constituted casting three reinforced concrete (RC) beams. The first beam was the control beam, the second beam was corroded, while the third beam was corroded and retrofitted with a single bamboo laminate at the tensile underside of the third beam. The dimensions of all the specimens were identical. Experimental data on deflection and the load of each beam were observed and recorded. The disparity in yield, and ultimate load capacity was investigated.

**Figure 1:**
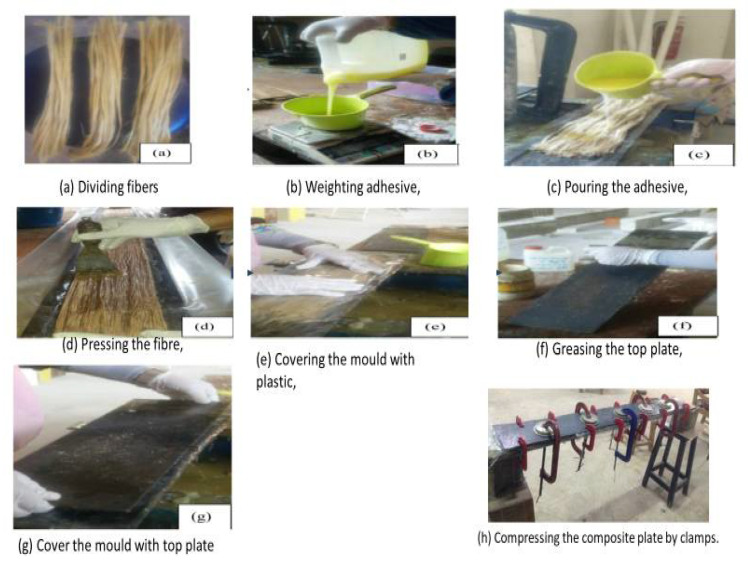
Process of fabricating bamboo fiber laminate.

Two-part epoxy and hardener served as the polymer matrix component in the BFRP laminate. Epikote 816 resin manufactured by Hexion, London, United Kingdom, and Epikure F205 (manufactured by Kian Resin Chemical Co., Birjand, Iran) mixed with a 2:1 ratio by weight according to the manufacturer’s specification were utilized. Sikadur 30 LP adhesive manufactured by SIKA corporation, New Jersey, USA, was used to bond the BFRP laminate to the RC beam. The Sikadur adhesive was prepared by blending its two parts (resin and hardener) with a 2:1 ratio. Table 1 shows the mechanical properties of Sikadur 30 LP cured as provided by the manufacturer. As illustrated in Figure 1, the process of developing bamboo fiber laminate entails: Dividing fibers, applications of adhesives on fibers, compressing fibers to the required thickness. The properties of the bamboo fiber are: tensile strength (520 MPa), elongation (2.3%), Young’s modulus (32 GPa), moisture content (7.79 wt.%), density(1.4 g/cm^3^), and moisture absorbency (8.7%).

The developed bamboo laminate was fabricated with both 35% and 45% fiber contents by mass. The tensile strengths are as follows—35% fiber laminate (72.8 MPa) and 45% fiber laminate (99.3 MPa).

**Figure 2:**
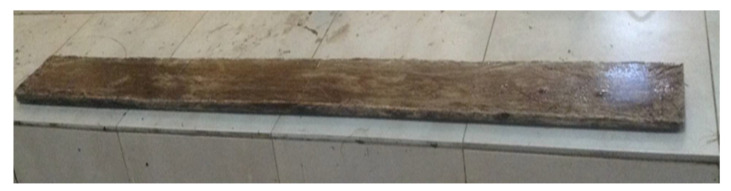
Fabricated bamboo laminate.

The fabricated bamboo fiber laminate is shown in Figure 2. The contact surfaces of both the RC beam and the bamboo laminate were roughened mechanically with a hand grinder to ensure proper adhesion and prevent premature debonding. A coat of adhesive was applied on the surface of the beam, after which the laminate was placed on the adhesive, and pressure was applied to it for about 24 h.

FRP wrapping on the beam was a one-sided wrap and continuous along the span of the length of the beam.

Timber formworks were fabricated using plywood sheets. After the mold fabrication, a low-density polymer termed nylon was applied to the mold walls to avoid its adhesion with cured concrete. The steel bars were then be cut to the required length, bent to the required shape, then tied together, forming the reinforcement cage. The reinforcement cage was then be placed inside the timber mold, ensuring that the desired concrete cover is achieved. The prepared concrete was then poured into the mold and allowed to set. After 24 h, the three beams were demolded and placed in a curing tank for 28 days at 20 °C. The concrete employed to cast the beam specimens was also used to cast three—150 mm concrete cube specimens to determine its 28-day compressive strength based on the BS EN, 12390-3 [34] procedures. The study adopted a mix proportion for a nominal mix of grade M25 concrete representing 1:1:3 (one part cement, one part sand, and three parts of granite) with a water-cement (W/C) ratio of 0.6. The concrete mixing and production followed standard procedures [35].

### Experimental Program

**Figure 3:**
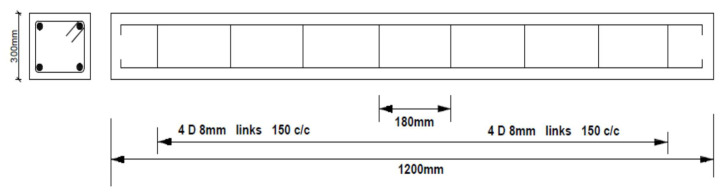
Beam reinforcement details.

Each rectangular beam was 300 mm × 200 mm in cross-section and 1200 mm long. The steel reinforcement consisted of two high yield 16 mm diameter steel bars at the bottom and two 12 mm diameter bars at the top. The shear reinforcements consisted of 10 mm diameter bars 150 mm center to center. The clear concrete cover was 25 mm on all sides of the beam specimens. The second and third beams had their reinforcements protruded out of the concrete to establish electrical connections to induce accelerated corrosion. A section of the beam showing reinforcement details is presented in Figure 3.

**Figure 4:**
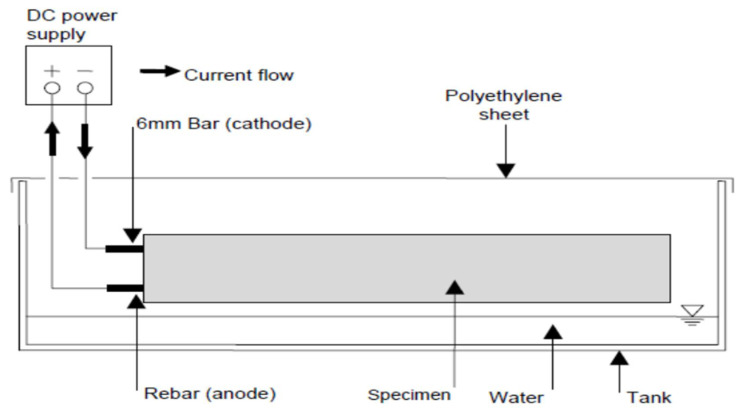
Schematic of accelerated corrosion set-up.

**Figure 5:**
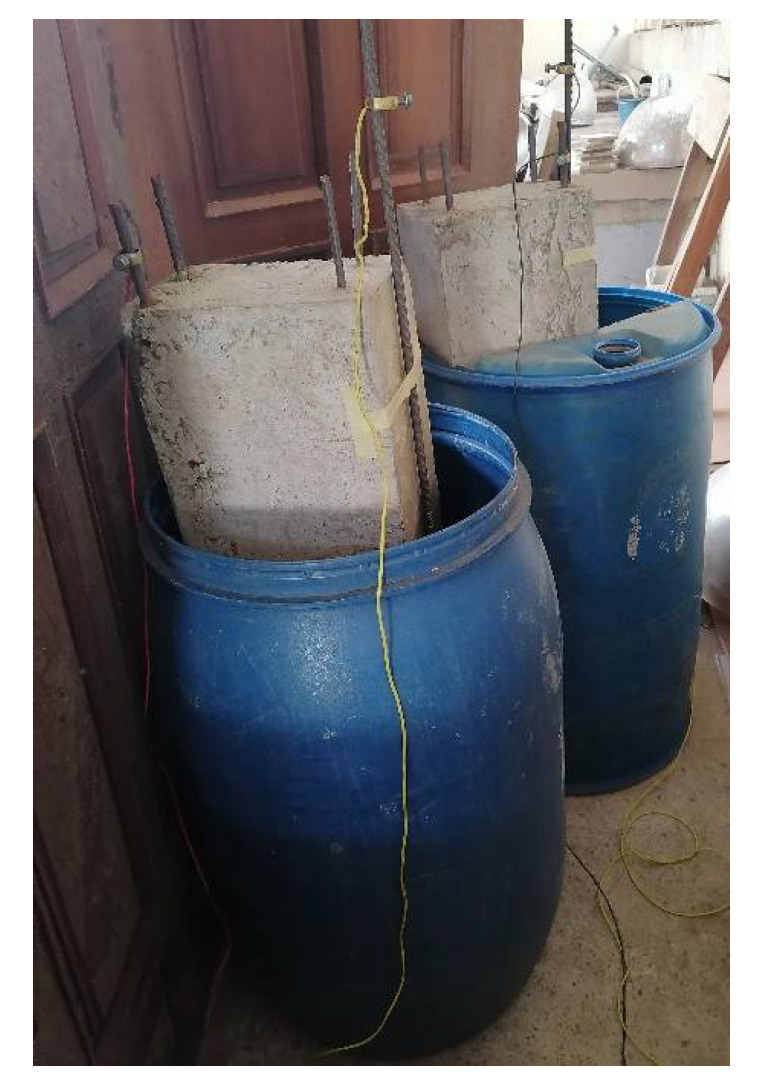
Simulated corrosion set-up in the laboratory.

Two beams were immersed to 75% of their length in a tank containing 6% of industrial salt solution (NaCl), which was subjected to wet and dry cycles (three weeks wet and one week dry alternately in total). This method is a combination of both the impressed current technique and alternate wetting and drying technique. The beams were subject to a constant electric current that was applied during the wetting period. The mass-loss rate of tensile bars is regarded as corrosion rate and will be estimated employing using Faraday’s law. The equation expressing mass loss is represented by:

(1) $$m=\frac{atI}{\left[nF\right]}\;$$

where *m* = mass loss, *a* = atomic mass of iron (55.85 gr), *t* = corrosion process time, *I* = corrosion current, *n* = reacting electrode valence for the material (which for steel is 2), and *F* = faraday’s constant (96,500 C/mol). Figure 4 and Figure 5 show the schematic of accelerated corrosion set-up and simulated corrosion set-up in the laboratory, respectively.

**Figure 6:**
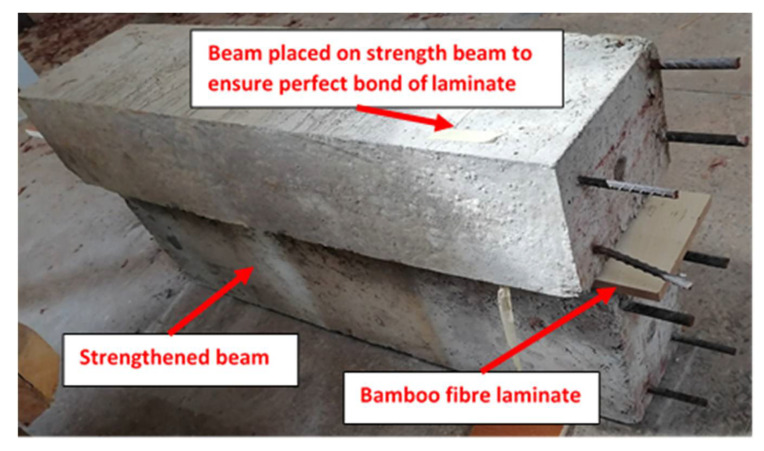
Bonding of bamboo laminate to the surface of the corroded beam.

All the specimens were tested in a 50 tons universal testing machine (UTM), manufactured by Dongguan HongTuo Instrument Co.,Ltd., GuangDong, China. After the 28-day curing at 20 °C was done, the beams were washed, and their surface was cleaned and checked for cracks. The second and third beams were then immersed about 2/3 of a 200 L drum containing 0.6 mol NaCl solution, which was then left for a week. The tensile reinforcements served as the connecting point for the anode, while an external reinforcement served as the cathode. Alternate wetting and drying were also used in inducing accelerated corrosion to save energy costs for three weeks with two weeks of wetting and a week of drying. Figure 6 presents a corroded beam and the process of gluing the laminate to its surface.

**Figure 7:**
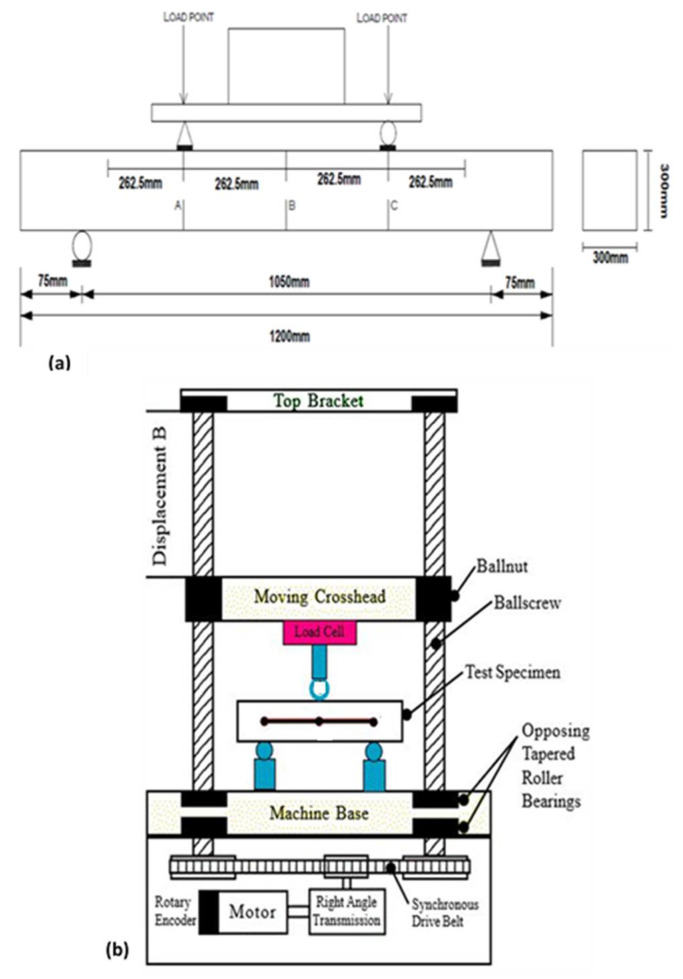
(**a**) Loading details of bending test on the control and corroded beam; (**b**) schematic arrangement of testing equipment.

**Figure 8:**
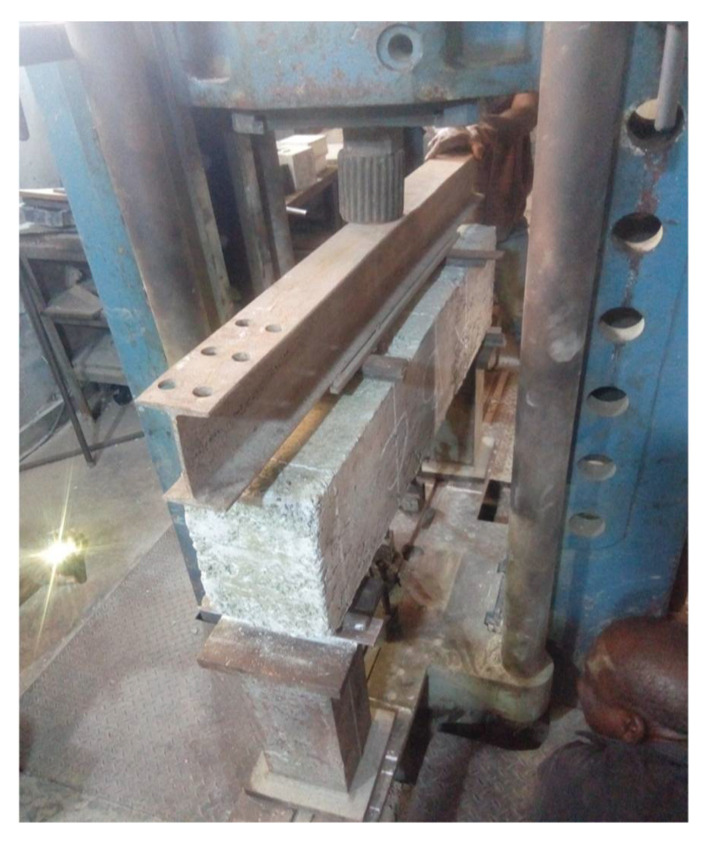
Testing of a beam using an ultimate testing machine.

During testing, the beams were mounted on steel supports with a clear span of 1050 mm. The load was then applied with a 50 ton UTM at 1 ton increments. The total applied load was a combination of the weight of the spreader beam and the load applied from the UTM. Three dial gauges were placed below the beam at the midspan and below the load application points to monitor the beam’s deflections. Deflections were recorded after each load increment. Figure 7a shows the schematic setup, Figure 7b shows the schematic arrangement of the testing equipment, and the process of testing a beam using ultimate testing machine is presented in Figure 8.

## 3. Results and Discussion

The ultimate load-carrying capacity of all the beams and the nature of the failure are given in Table 2. It shows that for the corroded un-strengthened beam, its deflection is 18.6 mm, which is less than the control beam. The retrofitted or strengthened corroded beam has a deflection of 22.7 mm, which is less than the control beam but higher than the corroded un-strengthened beam. The margin of structural capacity demonstrated by the retrofitted corroded member was evidence that bamboo laminate has somewhat beneficial effect in retrofitting distressed members. As such, the service life of weak members can be extended with natural materials.

Table 2 summarizes the results of the experiments corresponding to the beam samples and loading schemes: Load at the first crack (K), flexural load at the first crack (F) (occurring at the tension face), maximum load (Fu), the increase of the bearing capacity due to laminate application (Dexp), the mid-span deflection at maximum load δmax, and the decrease of deflection compared to the level of the control beam (Ddispl).

### 3.1. Load-Deflection

#### 3.1.1. Control Beam

**Figure 9:**
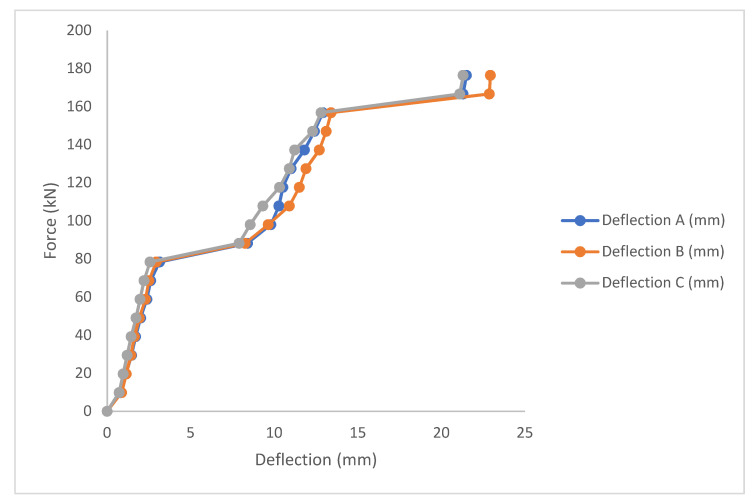
Load deflection plot for control beam.

The load-deflection curve for the control beam includes a linear response of load in the range between 65–80 kN. The first apparent crack was seen at a load of 84 kN. After 120 kN, flexural load cracks showed and widened as loading increased. The maximum load was 180.4 kN, as shown in Figure 9. The control beam showed the most ductility as the steel reinforcements inside the beam was not corroded and were in good condition after the beam was opened and the steel removed for corrosion mass loss calculation. The deflection at the three points of measurement (dial gauges) were highest for the control beam. The control beam had some moment capacity beyond the yield point, but this capacity was not detected from the experiment, as the UTM used was graded in 1 ton (9.8 kN) increments. From the load-deflection curve, there are three phases for the beam. In the first phase, the control beam’s stiffness is maintained. In the second phase, where the beam cracks see the stiffness of the beam reduce, while at the last stage, the mid-span deflection increases from 12.9–21.3 mm with an increase of 11 kN of load force.

#### 3.1.2. Corroded Un-Strengthened Beam

**Figure 10:**
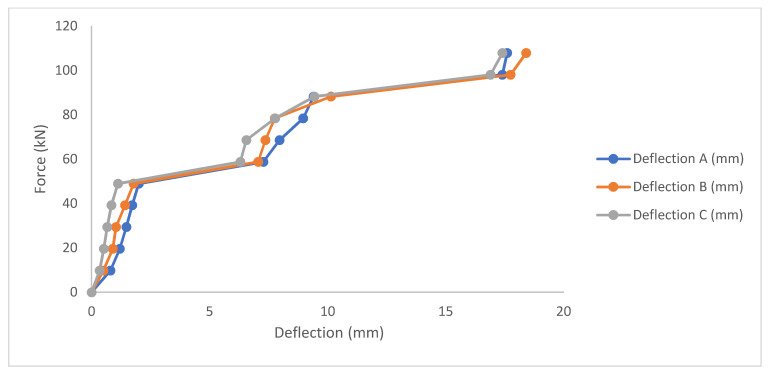
Load deflection plot for corroded un-strengthened beam.

Figure 10 shows the load deflection plot for corroded un-strengthened beam. The same pattern of curve was also shown for the corroded beam as the control. The difference was shown to be that its ranges of value been lesser than the corroded beam. This can be attributed to the corrosion of the reinforcement as this reduced the deflection capacity and the yield. Here the first sign of crack was at 59 kN. Flexural crack followed subsequently after 68 kN. After the maximum load, the beam behavior could not be observed as the UTM machine gauge returns back to zero after maximum load, hence the reason for the graph curve stopping at peak level. The maximum load was observed to be at 113.76 kN.

#### 3.1.3. Retrofitted Corroded Beam

**Figure 11:**
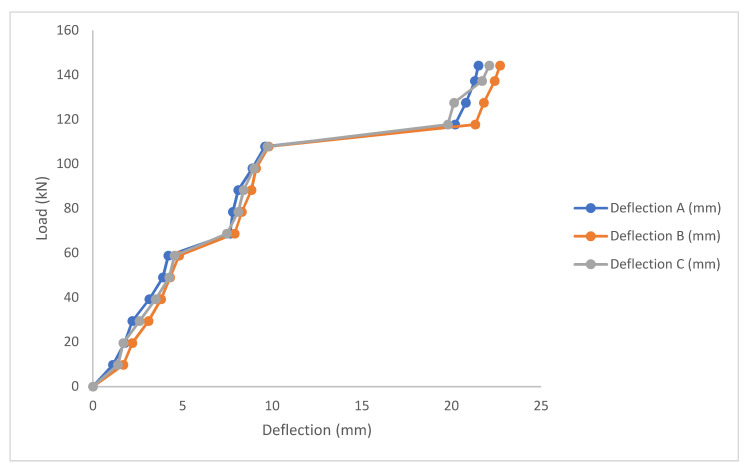
Load deflection plot for bamboo laminate corroded beam.

Figure 11 shows the load deflection plot for bamboo laminate corroded beam. Using a single bamboo laminate in the tensile region increased the beam capacity up to 21.1% greater than the corroded beam. Though 20% lesser than the control beam, this can be attributed to mass loss of the steel rebar. The curve repeated the same pattern with this sample, with the first appearance of a crack appearing at 68.6 kN. The flexural crack began appearing after 98 kN, and the maximum load is at 144.16 kN. The mid-span deflections for the laminate sample were also bigger when compared to the corroded un-strengthened sample, with the mid-span deflection at maximum (22.7 kN) being slightly similar to the control beam (22.93 kN).

**Figure 12:**
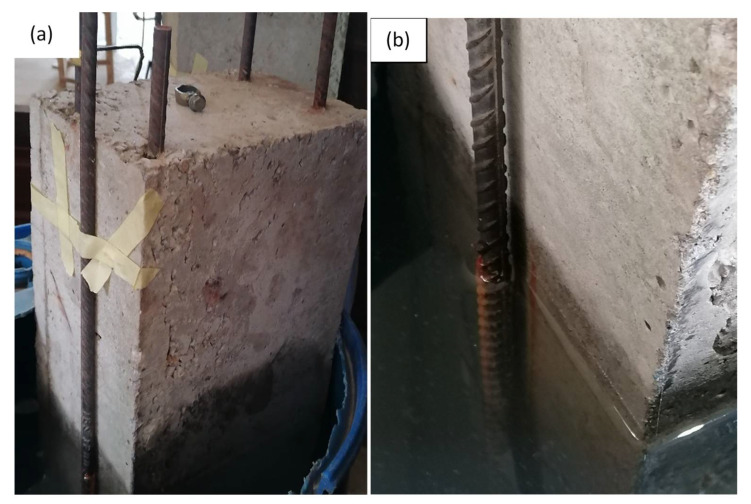
Evidence of corrosion on beams: (**a**) At early stage; (**b**) at maturity.

Evidence of corrosion on beams is presented in Figure 12. After the beam had undergone flexural testing, the beam was demolished, and three samples each for both the 16 and 12 mm rebar were removed, cut to a length of 550 mm, and then weighed. This was done for the three beam samples.

However, due to combining two methods of corrosion acceleration process, namely cyclic wet and drying with the impressed current technique, the mass loss was derived by comparing the final weight or mass of the tensile steel with the original weight of the steel. The control beam was used as a reference and the weight were compared to determine the mass loss. Weight variation and mass loss in embedded reinforcement bars in beams is presented in Table 3, and Table 4 shows the statistical description of mass loss data.

### 3.2. General Comparative View of the Tested Beam Samples

As shown in the results, the beam laminate sample performed slightly better than the corroded beam in terms of strength and deflection but less than the control beam. Clearly, the condition of the steel has a significant role in the sample’s strength and deflections. The bamboo laminate was responsible for the corroded retrofitted beam’s strength and deflection increase, as can be deduced from the results.

### 3.3. Comparisons to Parallel Research

Most research on retrofitted beams dealt with various failure modes from flexural failure, concrete cover separation, and interfacial debonding [25,36]. Chin et al. [22] research on the strengthening of RC beams in flexure with bamboo plates showed that retrofitting increased the beam’s original capacity up to 98% of the control beam. Additionally, the research showed that the bamboo plates managed to alleviate and change the failure mode from brittle to more ductile behavior. This ductile behavior was also observed for the corroded retrofitted beam as nearly as ductile as the control beam. Due to the UTM machine dial gauge inability to crush beyond maximum load, the failure mode of the retrofitted beam could not be observed with absolute certainty. However, the signs of interfacial crack debonding were seen for the retrofitted beam as the bamboo laminate had a rupture in its middle.

Hafizah et al. [25] research also showed that retrofitting beams with natural fibers increases beam deflection and stiffness, which is similar to the findings by Chin et al. [22]. This was confirmed by this research also, as deflections for the corroded retrofitted beams increased by 22% compared to the un-strengthened corroded beam.

Most beams designed to fail in flexure fail in flexure bending, except for retrofitted beams which exhibit a totally different failure mode, mostly cover debonding [22,25]. Hwoever, this research was different in terms of its retrofitted beam failure mode. It failed by interfacial cracking debonding, which could result from the corrosion of the steel reinforcements.

The level of corrosion-induced for 33 days in this research was significantly lower compared to the level of corrosion employed by Banu et al. [3], whose research showed that 33 days of the corrosion inducement caused 20% of mass loss in steel reinforcements. The reason for this could be attributed to the cathode bar being an external component from the beam, which implies that the corrosion loss would be shared by all four reinforcements, including the stirrups in the beam samples.

## 4. Conclusions

This study focused on the structural retrofitting of corroded reinforced concrete beams using bamboo fiber laminate. The following conclusions were drawn from the study:

- The retrofitting capacity of bamboo laminate was evidently demonstrated. The bamboo laminate increased the ductility and strength of the corroded beam, thus aiding the load resistance of the beam. The retrofitted beam exhibited strength somewhat close to that of the control beam. Thus, it is practically shown that bamboo fiber laminate has significant structural application on distressed elements.
- The control beam and corroded un-strengthened beam failed by way of a flexural failure, while the bamboo-strengthened beam exhibited cracking debonding. The sudden change in the cross-section at the end of the laminate contributed to this.
- The corroded un-strengthened beam exhibited the highest number of cracks, with the control beam exhibiting the lowest number of cracks. Again, the impact of the fabricated laminate is demonstrated by the delay of cack development in the distressed member.

## Figures and Tables

**Table 1:** Mechanical properties of Sikadur 30 LP cured at 25 °C.

Property	Value
Compressive strength	>85 MPa
Tensile strength	14–17 MPa
Tensile modulus	~10 GPa
Flexural strength	20–25 MPa
Tensile adhesion strength (concrete)	>4 MPa
Pot life	~60 min
Curing time	7 days

**Table 2:** Comparisons between maximum load and deflections.

Beam Samples	K (kN)	F (kN)	F_u_ (kN)	D_exp_ (%)	δ_max_ (mm)	D_displ_ (%)	Nature of Failure
Control beam	84	98	180.4	-	22.93	0	Flexure failing
Corroded un-strengthened beam	59	68	113.76	-	18.6	18.8	Flexure failure
Retrofitted corroded beam	68.6	98	144.16	26.7	22.7	1	Interfacial crack debonding

**Table 3:** Steel reinforcement sample weight and mass loss.

Sample Name	Sample Diameter (mm)	Sample Weight (Kg)	Sample Weight Average (Kg)	Mass Loss of Tensile Steel (%)
Control beam rebar				
	12	0.35	0.35	0
	12	0.35		0
	12	0.35		0
	16	0.55	0.55	0
	16	0.55		0
	16	0.55		0
Corroded beam un-strengthened with bamboo laminate				
	12	0.34	0.33	2.86
	12	0.33		5.71
	12	0.32		8.57
	16	0.53	0.53	3.64
	16	0.53		3.64
	16	0.54		1.82
Corroded beam rebar strengthened with bamboo laminate				
	12	0.34	0.33	2.86
	12	0.33		5.71
	12	032		8.57
	16	0.54	0.53	1.82
	16	0.53		3.64
	16	0.53		3.64

**Table 4:** Statistical analysis of mass loss data.

	N Total	Mean	Standard Deviation	Sum	Minimum	Median	Maximum
A	18	2.91556	2.81876	52.48	0	2.86	8.57

## Data Availability

No new data were created or analyzed in this study. Data sharing is not applicable to this article.
